# Production of complex viral glycoproteins in plants as vaccine immunogens

**DOI:** 10.1111/pbi.12963

**Published:** 2018-07-06

**Authors:** Emmanuel Margolin, Ros Chapman, Anna‐Lise Williamson, Edward P. Rybicki, Ann E. Meyers

**Affiliations:** ^1^ Division of Medical Virology Department of Pathology Faculty of Health Sciences University of Cape Town Cape Town South Africa; ^2^ Institute of Infectious Disease and Molecular Medicine Faculty of Health Sciences University of Cape Town Cape Town South Africa; ^3^ Biopharming Research Unit Department of Molecular and Cell Biology University of Cape Town Cape Town South Africa

**Keywords:** glycoprotein, biopharming, vaccine, chaperone

## Abstract

Plant molecular farming offers a cost‐effective and scalable approach to the expression of recombinant proteins which has been proposed as an alternative to conventional production platforms for developing countries. In recent years, numerous proofs of concept have established that plants can produce biologically active recombinant proteins and immunologically relevant vaccine antigens that are comparable to those made in conventional expression systems. Driving many of these advances is the remarkable plasticity of the plant proteome which enables extensive engineering of the host cell, as well as the development of improved expression vectors facilitating higher levels of protein production. To date, the only plant‐derived viral glycoprotein to be tested in humans is the influenza haemagglutinin which expresses at ~50 mg/kg. However, many other viral glycoproteins that have potential as vaccine immunogens only accumulate at low levels *in planta*. A critical consideration for the production of many of these proteins in heterologous expression systems is the complexity of post‐translational modifications, such as control of folding, glycosylation and disulphide bridging, which is required to reproduce the native glycoprotein structure. In this review, we will address potential shortcomings of plant expression systems and discuss strategies to optimally exploit the technology for the production of immunologically relevant and structurally authentic glycoproteins for use as vaccine immunogens.

## Introduction

The development of plant‐based expression systems for the scalable and economical production of recombinant proteins constitutes a major paradigm shift in the manufacturing of pharmaceuticals. This technology is fast becoming established in several niche areas where traditional manufacturing approaches fall short—such as where a rapid response is required, or where production scalability is limited (Rybicki, 2009; Stoger *et al*., 2014; Streatfield *et al*., 2015). A major driving force for molecular farming is the lower costs involved; this is for both the production of biomass and the less stringent infrastructure requirements than for typical fermentation systems (Edgue *et al*., 2017; Rybicki, 2009; Tschofen *et al*., 2016). These unique features are particularly appealing for developing countries which typically suffer a greater burden of infectious disease, and where the infrastructure for traditional pharmaceutical production is often largely or completely absent (Hefferon, 2013; Ma *et al*., 2013; Rybicki *et al*., 2013). In addition, plants are safer than traditional expression systems as they do not produce endotoxins or support the growth of potentially infectious viruses or prions (Moustafa *et al*., 2016). Plants can also mediate complex post‐translational modifications, including both glycosylation and disulphide bond formation (Faye *et al*., 2005; Tschofen *et al*., 2016). Furthermore, the remarkable plasticity of the plant proteome enables the simultaneous co‐expression of multiple proteins, enabling the carefully regulated manipulation of the secretory pathway for the production of proteins with homogenous mammalian‐like glycosylation (Castilho and Steinkellner, 2012; Castilho *et al*., 2010). Lastly, plant‐derived vaccine antigens offer the potential for oral immunization, although it is expected that some degree of formulation would be necessary at the least to ensure dose consistency (Rybicki, 2009).

**Figure 1:**
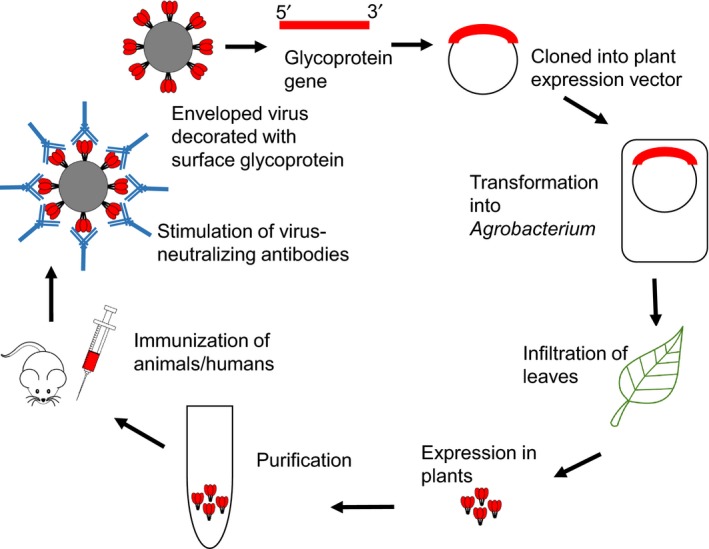
Production of recombinant glycoproteins as subunit vaccines to protect against viral infections. Virus‐derived glycoproteins often represent the major antigenic determinants of viral infections, and therefore, the production of recombinant glycoproteins in heterologous expression systems is of considerable interest for vaccine development. This diagram shows a typical enveloped virion where the surface is decorated with trimeric glycoproteins which facilitate entry into susceptible target cells. The glycoprotein gene is cloned into a heterologous expression vector and the protein overexpressed (for example, transiently by *Agrobacterium*‐mediated infiltration). The glycoprotein is purified from contaminating host proteins and formulated for immunization to elicit neutralizing antibodies against the virus.

Immunization is unarguably the most effective biomedical intervention against infectious diseases and has resulted in the successful licensing of over 70 vaccines for use in humans (Nabel, 2013). The most consistent biomarker of vaccine efficacy is the induction of neutralizing antibodies, which in the case of many enveloped viruses target the glycoprotein spikes on their surfaces (Nabel, 2013). Given this observation, the development of glycoprotein‐based antigens is a major focal point in the development of vaccines against many viral pathogens (summarized in Figure 1) (Hajj Hussein *et al*., 2015). Although not a focus of this review, it is also noteworthy that, in addition to their use as vaccine antigens, recombinant glycoproteins also represent potential diagnostic reagents, particularly in the One Health initiative (Rybicki, 2017).

Whilst recombinant viral structural proteins generally accumulate at reasonable levels in plants, many viral glycoproteins express poorly, or even below, the threshold of detection by standard methods, for reasons that are not completely understood. This has been reported for a number of different viral glycoproteins and has most likely been observed for others in work that has not been published. In our experience, this is associated with severe tissue necrosis that is indicative of endoplasmic reticulum (ER) stress arising from the accumulation of misfolded proteins and the associated unfolded protein response (Howell, 2013; Pera *et al*., 2015; Phoolcharoen *et al*., 2011). This phenomenon has been described in the literature for the extracellular subunit of the Ebola glycoprotein (GP1), rotavirus VP7 (Pera *et al*., 2015; Phoolcharoen *et al*., 2011) and observed in our laboratory for the full‐length GnGc proteins of both Rift Valley fever virus and Crimean–Congo haemorrhagic fever virus, as well as the premembrane and envelope proteins of Zika virus. It is worth noting, however, that tissue necrosis is not limited to viral glycoproteins and may arise from the overexpression of any protein that exceeds the folding capacity of the system (Liu and Howell, 2010).

Although a minimum expression yield of 1% of the total soluble protein is generally regarded as a prerequisite to justify industrial production, this is rarely achieved and especially not for viral glycoproteins (Rybicki, 2009). However, many of the influenza haemagglutinin (HA) glycoproteins express at unusually high levels *in planta,* and candidate vaccine antigens have been reported to accumulate at ~50 mg/kg (D'Aoust *et al*., 2008; Shoji *et al*., 2011). Amongst the most impressive demonstrations of plant molecular farming is the pioneering work of Medicago Inc. which have developed a highly scalable expression platform for the production of the influenza virus glycoprotein. Their lead vaccine candidate, plant‐derived influenza virus haemagglutinin (HA) virus‐like particles, is currently being evaluated in phase 3 efficacy trials which, if successful, should lead to FDA approval by 2020. At the time of writing, influenza HA remains the only viral glycoprotein expressed in plants that has been evaluated in human trials (Pillet *et al*., 2016).

It is unclear why many viral glycoproteins only accumulate at low levels in *planta* although we have surmised that this may be due to either incompatibility with the endogenous plant chaperones or inadequate levels of crucial chaperones required to facilitate folding of the overexpressed protein. The lack of adequate levels of molecular chaperones has been acknowledged as a bottleneck in other heterologous expression systems (Hsu and Betenbaugh, 1997; Rudolph and Lilie, 1996). If this is taken to be true for plants, a major challenge which will need to be addressed is the identification of which chaperone or even combination of chaperones is rate‐limiting for the expression of any given glycoprotein. In this review, we will discuss recent successes in the production of plant‐produced viral glycoproteins and critically discuss considerations for the production of authentic, vaccine‐relevant mimetics of native glycoproteins in plants.

## Factors influencing expression of functional glycoproteins

Despite the well‐documented successes in certain instances of expression of virus glycoprotein vaccine candidates in plants, in many cases, yields are too low for generating sufficiently large batches for vaccination programmes. However, there are a number of different strategies that can be taken into consideration which may not only improve yields, but could also improve functionality of the antigens. These include factors such as targeting proteins to the secretory pathway, N‐glycosylation, proteolytic maturation along the secretory pathway and the influence of chaperones in the endoplasmic reticulum.

### Targeting recombinant glycoproteins into the secretory pathway

In eukaryotes, the translocation of glycoproteins through the ER is essential to impart authentic glycosylation and other post‐translational modifications (PTMs), as well as to impose quality control prior to further progress along the secretory pathway. In the ER, newly synthesized proteins interact with molecular chaperones and glycosylation enzymes, which act in concert to facilitate the folding and maturation of the protein (Braakman and Hebert, 2013). Terminally misfolded proteins or incompletely assembled polypeptides do not progress beyond the ER but instead are targeted into the ER‐associated protein degradation (ERAD) pathway for proteosomal degradation (Hiller *et al*., 1996; Jensen *et al*., 1995; Lippincott‐Schwartz *et al*., 1988; Sommer and Jentsch, 1993; Ward *et al*., 1995). Given the critical role of the secretory pathway to facilitate proper folding and glycosylation, targeting recombinant proteins to different subcellular localizations may improve their accumulation at the expense of the native structure.

Glycoproteins naturally encode an N‐terminal signal sequence (typically 20–30 amino acid residues) that targets the newly synthesized protein into the ER (Hegde and Bernstein, 2006). The nature of this signal peptide determines the efficiency of translocation into the ER and subsequent cleavage of the leader peptide from the glycoprotein (Braakman and Hebert, 2013). To illustrate this point, it has been reported that in mammalian cells the native HIV Env signal peptide is inefficiently cleaved, resulting in prolonged retention of the protein in the ER and contamination of virions with aberrantly folded Env species (Crooks *et al*., 2011; Land *et al*., 2003; Li *et al*., 1996, 2000). The use of highly efficient foreign signal peptides is often exploited for the production of glycoproteins in heterologous systems, such as the honeybee melittin signal sequence in insect cells or the tissue plasminogen activator leader sequence in mammalian expression systems (Sanders *et al*., 2013; Tessier *et al*., 1991). Several examples of heterologous signal peptides used for glycoprotein expression in plants are listed in Table 1. We routinely make use of the LPH leader sequence, a murine monoclonal antibody‐derived signal peptide, to target recombinant proteins into the plant secretory pathway. This approach has shown promise for the production of trimeric Env glycoproteins from HIV and of a soluble Gn antigen from RVFV (Mbewana, 2017).

**Table 1:** Notable examples of heterologous signal peptides used for the production of viral glycoproteins in plants

Signal peptide	Origin of signal peptide	Antigen	Expression host	Reference
LPH	Signal peptide from murine monoclonal antibody mAb24 heavy chain (Fischer *et al*., 1999)	Rift Valley fever virus Gn	*N. benthamiana*	Mbewana (2017)
		Chimeric Gn/influenza HA particle‐forming fusion protein	*N. benthamiana*	Mbewana (2017)
		HIV Env gp140	*N. benthamiana*	Margolin *et al*. (submitted for publication)
PDI	Signal sequence from protein disulphide isomerase of *Medicago sativa* GenBank CAA77575.1	Influenza H1 HA	*N. benthamiana*	D'Aoust *et al*. (2008)
		Chimeric HIV gp140/influenza HA particle‐forming fusion protein	*N. benthamiana*	WO 2012/083 445 A1
PR‐1a	Signal peptide derived from pathogenesis‐related protein 1a of *Nicotiana tabacum* NCBI BAA14220	Influenza H5	*N. benthamiana*	Shoji *et al*. (2009a)
		Yellow fever E protein	*N. benthamiana*	Tottey *et al*. (2018)
Barley α‐amylase	Signal peptide from barley α‐amylase GenBank CAX51374	Truncated HIV‐1 gp41	*N. benthamiana*	Kessans *et al*. (2013)
		SIV gp130	*Zea mays*	Horn *et al*. (2003)
PR‐S	Signal peptide from extracellular tobacco protein PR‐S (Sijmons *et al*., 1990)	Rabies G protein	*N. tabacum*	Ashraf *et al*. (2005)

TSP, total soluble protein.

Another commonly used signal peptide is the protein disulphide isomerase (PDI) signal sequence which was used by Medicago Inc. for the production of recombinant influenza H1 HA, but not H5 HA which was expressed under the control of the native signal peptide (D'Aoust *et al*., 2008). In contrast, Fraunhofer USA has reported the use of the pathogenesis‐related protein (PRP)‐1a signal peptide to produce soluble influenza H5 HA in plants (Shoji *et al*., 2009a). We have found that a soluble RVFV Gn glycoprotein was only expressed when fused to the LPH signal sequence but not the PDI signal sequence (Mbewana, 2017). Other pertinent examples include the rabies virus glycoprotein which has been produced in transgenic tomatoes using the native signal peptide and in transgenic *Nicotiana tabacum* under the control of the PRP signal peptide (Ashraf *et al*., 2005; McGarvey *et al*., 1995). The latter antigen, which was further modified to contain a C‐terminal ER retention signal, protected mice against lethal rabies challenge (Ashraf *et al*., 2005).

It appears that the impact of different signal peptides on heterologous protein expression can only be determined empirically. To illustrate this point further, it was reported that detectable expression of a truncated HIV gp41 antigen could be achieved in tobacco plants using the barley alpha‐amylase signal peptide, but not when the signal sequences from apple pectinase, *N. plumbaginifolia* calreticulin or rice alpha‐amylase were used (Kessans *et al*., 2013). A recent study has suggested that tissue necrosis during heterologous protein expression *in planta* could be linked to improper cleavage of the signal peptide. The authors reported that substituting the *Arabidopsis thaliana* chitinase signal peptide for the native signal sequence of transforming growth factor‐β1 resulted in high levels of protein production whilst still maintaining a healthy phenotype in *N. benthamiana* plants (Wilbers *et al*., 2016).

It is also worth noting that the targeting of the recombinant H5 HA protein into the plant secretory pathway resulted in the formation of enveloped VLPs which budded from the plasma membrane in the absence of other accessory proteins (D'Aoust *et al*., 2008). This phenomenon appears to be unique to plants, as other heterologous expression systems require neuraminidase to sever the sialic acid linkages that tether budding virions to the host cell (Chen *et al*., 2007). Plants, however, lack endogenous sialic acid activity, enabling HA‐containing particles to bud from the plasma membrane into the apoplastic spaces between plant leaves (D'Aoust *et al*., 2008; Seveno *et al*., 2004). Our group has also observed the assembly of similar enveloped H5 HA VLPs (Rybicki 2014), although early attempts to recover the recombinant protein that included the use of Triton‐X100 precluded the recovery of enveloped VLPs (Mortimer *et al*., 2012). A similar phenomenon has been described by Fraunhofer USA for the production of enveloped Ebola virus VLPs (eVLP) composed of the full‐length glycoprotein (Karczewski and Ysibov, 2016). This is a useful way of improving the immunogenicity of small soluble proteins which may otherwise be poorly immunogenic (Bachmann and Jennings, 2010; Zabel *et al*., 2013).

### The potential impact of plant‐derived N‐glycosylation

**Figure 2:**
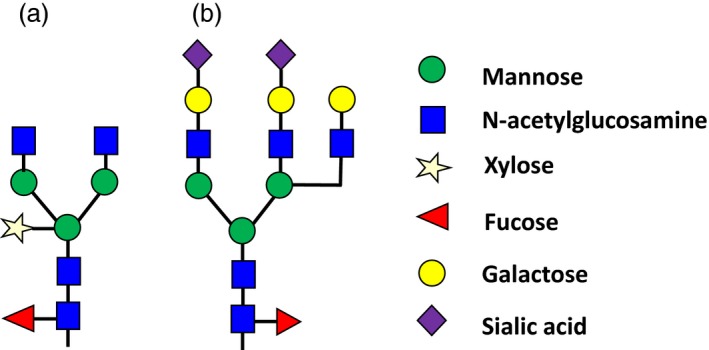
Comparison of typical N‐glycan structures from glycoproteins produced in (a) plants and (b) mammalian expression hosts. Whilst the core glycan machinery is preserved amongst eukaryotes, species‐specific modifications occur in the Golgi apparatus. Plant‐produced glycoproteins contain characteristic α‐(1,3) fucose and (β1,2) xylose residues, whereas mammalian‐derived proteins contain α(1,6)‐fucose moieties and terminal sialic acid residues. Sialylation does not occur naturally in plants.

Whilst the ability of plants to mediate N‐glycosylation is regarded as an advantage over more primitive prokaryotic expression systems, complex glycans derived from plants are unique in that they contain core β(1,2)‐xylose and α(1,3)‐fucose but lack both the β(1,4)‐galactose and sialic acid that is characteristic of mammalian cell glycosylation (Strasser *et al*., 2008; Figure 2). The observation that these glyco‐epitopes were immunogenic in small animals, and reports that serum antibodies against these epitopes can be found in humans, raised concerns of possible allergic reactions and rapid clearance following administration (Bardor *et al*., 2003; Bosch and Schots, 2010; Jin *et al*., 2008). In spite of this trepidation, Medicago's HA‐based vaccine candidates were reported to be safe, even in volunteers with pre‐existing plant allergies, suggesting that these concerns were largely unwarranted (Ward *et al*., 2014). However, it is acknowledged that some transient antibody responses to the plant‐derived glyco‐epitopes were observed, although these were generally weak (Ward *et al*., 2014). The potential negative implications for use of proteins with these plant‐specific glyco‐epitopes have prompted extensive efforts to humanize the glycosylation of proteins in plants by elimination of plant‐specific glycans and the introduction of human glycosyltransferases [reviewed by Strasser *et al*. (2014)]. It is also interesting to note that a number of different plant polysaccharides are even being explored as adjuvants, with several having advanced into clinical trials [reviewed by Rosales‐Mendoza *et al*. (2016)].

The HIV Env glycoprotein is amongst the most heavily glycosylated proteins described, with approximately half the molecular mass of gp120 being attributed to glycans (Joyce *et al*., 2016; Lasky *et al*., 1986). These glycans are host‐derived and are considered ‘self‐antigens’, enabling the virus to mask vulnerable epitopes with a poorly immunogenic shield (Jardine *et al*., 2013; Julien *et al*., 2013; McGuire *et al*., 2013; Sok *et al*., 2016; Wei *et al*., 2003). Similarly, influenza HA also contains multiple glycosylation sites which appear to have been increasing since 1918, presumably to enable the virus to escape from neutralizing antibodies (Herve *et al*., 2015; Job *et al*., 2013; Skehel *et al*., 1984; Wei *et al*., 2010). Despite reports that glycan occupancy may be lower in plant‐produced proteins than in other production platforms, Medicago's plant‐produced HA was reported to contain glycans at all six potential N‐glycan sites in the extracellular portion of the protein (Le Mauff *et al*., 2015; Ward *et al*., 2014). It is unclear if potential underglycosylation will have an impact on recombinant HIV Env glycoproteins expressed in plants which are expected to contain ~90 glycans on each trimer (Cao *et al*., 2017). Underglycosylation could potentially compromise the folding of the glycoproteins as N‐glycans are critical for the recruitment of the lectin chaperones: calnexin and calreticulin (Wada *et al*., 1991). Furthermore, in the case of HIV‐1 Env, many of the broadly neutralizing antibodies isolated from natural infection target epitopes that contain a glycan component suggesting that a recombinant immunogen should reproduce these structures (Crispin *et al*., 2018). Encouragingly, a recent publication by Castilho and colleagues has suggested the co‐expression of a *Leishmania major* oligosaccaryltransferase can be used to improve the glycan occupancy of plant‐derived proteins (Castilho *et al*., 2018). Furthermore, ongoing efforts to humanize the glycosylation of recombinant plant‐produced proteins have demonstrated the successful removal of plant‐specific N‐glycan moieties and the introduction of human glycosyltransferases *in planta* (Strasser *et al*., 2014). Although this approach has been developed primarily for the production of recombinant antibodies, it is expected that as the range of plant‐produced viral glycoproteins expands that similar approaches will be implemented (Kallolimath and Steinkellner, 2015). A particularly interesting aspect of this work will be to determine the impact on the antigenicity and immunogenicity of plant‐produced viral glycoproteins expressed in wild‐type and glycoengineered plants.

The HIV Env glycoprotein appears to be unique in that many of the epitopes targeted by broadly neutralizing antibodies in viruses isolated from natural infections incorporate a glycan component (Andrabi *et al*., 2015; Balla‐Jhagjhoorsingh *et al*., 2013; Blattner *et al*., 2014; Falkowska *et al*., 2014; Gorman *et al*., 2016; Julien *et al*., 2013; Kong *et al*., 2013; Lyumkis *et al*., 2013; Sanders *et al*., 2002; Scanlan *et al*., 2002; Sok *et al*., 2014; Trkola *et al*., 1996). Encouragingly, the recombinant HIV Env gp140 produced by Rosenberg and colleagues in *N. benthamiana* was reported to react with several prototype monoclonal antibodies including 2G12, which specifically targets a glycan‐dependent epitope on the outer domain of the glycoprotein (Rosenberg *et al*., 2013). Glycoprofiling of the purified HIV Env protein revealed predominantly high‐mannose‐type glycans, with 21.5% containing plant‐specific α(1,3)‐fucose and β(1,2)‐xylose residues (Rosenberg *et al*., 2013). In natural infections, the virion‐associated Env comprises of predominantly unprocessed oligomannose moieties (Bonomelli *et al*., 2011; Doores *et al*., 2010). This is considered an important hallmark of the native structure, as the steric constraints of the trimer limit access of the glycan processing enzymes along the secretory pathway (Bonomelli *et al*., 2011; Doores *et al*., 2010; Pritchard *et al*., 2015). Whilst it is encouraging that plant‐produced HIV Env antigens can reproduce the structure of complex, glycan‐dependent epitopes, a recent study has suggested that sialic acid residues may contribute to the epitopes targeted by broadly neutralizing antibodies (Andrabi *et al*., 2017). Given this observation, wild‐type plants may not be ideal for the production of authentic mimetics of the glycoprotein as sialylation does not naturally occur in plants (Seveno *et al*., 2004). Recent studies have reported the artificial engineering of sialic acid biosynthetic pathways in plants which could be used to address this potential limitation (Castilho *et al*., 2010, 2013; Kallolimath *et al*., 2016; Paccalet *et al*., 2007). In apparent contradiction to this, a study conducted by Kong *et al*. comparing the influence of expression system‐dependent glycosylation concluded that cells lacking sialic acid may improve the immunogenicity of recombinant HIV Env immunogens, though not necessarily for neutralizing antibody responses (Kong *et al*., 2010).

### Proteolytic maturation of viral glycoproteins along the secretory pathway

Many glycoproteins require proteolytic cleavage to assume their mature fusion‐entry conformation required for infection; therefore, the ability of a heterologous expression system to mediate authentic processing of the protein is an important consideration. The HIV Env glycoprotein, for example, undergoes proteolytic cleavage by furin and other subtilisin‐like proteases during its translocation along the secretory pathway (Decroly *et al*., 1994, 1996; Gu *et al*., 1995; Hallenberger *et al*., 1992; Vollenweider *et al*., 1996). Proteolytic maturation of the glycoprotein is necessary for it to assume its correct quaternary structure (Ringe *et al*., 2013). This has important implications for vaccine design, as cleavage‐defective Env trimers have been reported to assume aberrant conformations which induce antibodies against epitopes that are sterically inaccessible in the context of the functional virion‐bound trimer and are therefore unable to neutralize the virus (Ringe *et al*., 2013; Tran *et al*., 2014).

Given these observations, trimeric HIV Env immunogens are often produced in mammalian cell culture by replacing the native cleavage site with a hexa‐arginine motif and co‐expressing heterologous furin to promote optimal cleavage of the recombinant glycoprotein (Binley *et al*., 2002). Furin activity does not naturally occur along the plant secretory pathway, although a precedent exists for the co‐expression of the protease to produce functional human transforming growth factor β1 *in planta,* suggesting that theoretically it may be possible to produce cleaved HIV Env glycoproteins in plants (Wilbers *et al*., 2016). Another potential complication with the production of a proteolytically cleaved glycoprotein is the labile association between the respective subunits. In the case of cleaved HIV Env trimers, an artificial disulphide bond is typically introduced at the interface of gp120 and the ectodomain of gp41 to ensure that the two subunits remain associated after cleavage (Binley *et al*., 2000). During natural infection, in the absence of any stabilizing mutations between the subunits, the gp120 subunit may be shed leaving gp41 stumps on the surface of the virion, which are proposed to serve as immunological decoys (Moore *et al*., 2006). Accordingly, it is important that a HIV Env‐derived vaccine antigen should be stable and that the gp120 and gp41 components remain associated for maximum immunogenicity.

Recent studies in mammalian cell cultures have managed to circumvent this requirement for furin cleavage by employing a flexible glycine‐rich linker in place of the native HIV Env cleavage site (Georgiev *et al*., 2015; Sharma *et al*., 2015). These cleavage‐independent trimers have been reported to be authentic mimics of the virion‐bound trimer, to retain native‐like antigenicity and to elicit neutralizing antibodies in preclinical studies (Georgiev *et al*., 2015; Pauthner *et al*., 2017; Sharma *et al*., 2015). A similar approach has also been reported for the production of respiratory syncytial virus fusion glycoprotein in mammalian cells, where the furin cleavage site was replaced with a flexible linker. This suggests that this strategy may be broadly applicable to other viral Type I glycoproteins, such as Ebolavirus GP (Joyce *et al*., 2016). We have exploited this design strategy to produce soluble cleavage‐independent HIV‐1 Env trimers in plants, facilitating the first immunogenicity studies of trimeric plant‐produced HIV‐1 Env proteins (Margolin *et al*., submitted for publication).

The structurally analogous influenza virus HA glycoprotein also requires proteolytic cleavage for viral infectivity, resulting in structural rearrangements that expose the fusion peptide (Bullough *et al*., 1994; Owens and Compans, 1990). Cleavage of H_0_ (the immature precursor glycoprotein) is mediated by ubiquitous cellular proteases, including the endoprotease furin, and may even be mediated by the proteases of co‐infecting bacteria (Galloway *et al*., 2013; Steinhauer, 1999; Stieneke‐Grober *et al*., 1992). The expression of HA in plants, however, is reported to yield a single product of 72 kDa, consistent with the size expected for uncleaved HA (D'Aoust *et al*., 2008; Mortimer *et al*., 2012; Shoji *et al*., 2011). A subsequent report confirmed the presence of low levels of HA_1_ and HA_2_ (the extracellular and transmembrane subunits of HA, respectively) suggesting that some level of precursor cleavage does occur, albeit very inefficiently (Le Mauff *et al*., 2015). Unlike with HIV, cleavage is not a requirement to elicit protective immunity against the influenza glycoprotein, as uncleaved H5 HA VLPs confer protection against lethal challenge (D'Aoust *et al*., 2008; Mett *et al*., 2008; Pillet *et al*., 2015; Shoji *et al*., 2009a).

### The chaperone folding pathway in the plant endoplasmic reticulum

**Figure 3:**
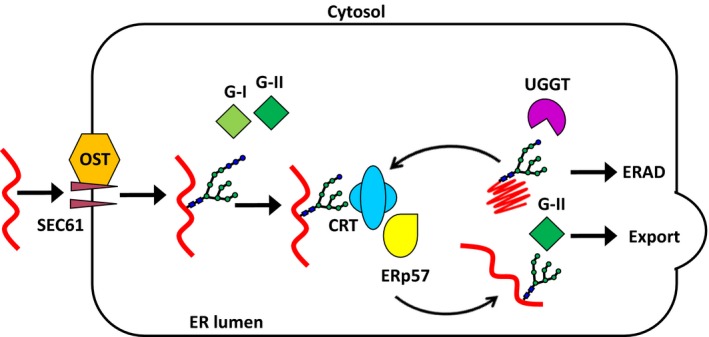
Carbohydrate‐mediated folding in the endoplasmic reticulum. Following translation, the nascent protein (red) enters into the endoplasmic reticulum (ER) through a translocon pore (SEC61) where it sequentially interacts with a network of chaperones and enzymes to acquire its correct higher order structure. During entry into the ER, the oligosaccharyltransferase complex (OST) transfers a preformed Glc_3_Man_9_ NAc_2_ glycan precursor to the Asn of the Asn‐X‐Ser/Thr motif in the polypeptide chain. The two outermost glucose residues are then sequentially removed by glucosidase I (G‐I) and glucosidase II (G‐II) signalling entry of the resulting monoglucosylated (GlcMan_9_ NAc_2_) protein into the calnexin (CNX)/calreticulin (CRT) folding cycle. Calnexin is the membrane‐bound homologue of calreticulin and preferentially interacts with nascent proteins associated with the ER membrane (only calreticulin is indicated in this diagram for the sake of simplicity). This interaction with CNX/CRT facilitates the co‐ordinated activities of other chaperones such as the oxidoreductase ERp57 to further assist with folding. Incorrectly folded proteins are reglucosylated by UDP‐glucose:glycoprotein glucosyltransferase (UGGT) signalling their re‐entry into the CNX/CRT folding cycle to undergo another round of chaperone‐mediated folding. In contrast, correctly folded proteins are acted on by G‐II, resulting in the removal of their remaining glucose residue. This signals release from the CNX/CRT pathway enabling export of the protein into the Golgi. Glycoproteins may undergo several rounds of chaperone‐mediated folding to assume the correct quaternary structure before continuing along the secretory pathway. In contrast, terminally misfolded glycoproteins are targeted for proteolytic degradation via the endoplasmic reticulin‐associated degradation (ERAD) pathway.

The endoplasmic reticulum in eukaryotes is a major site of protein folding and quality control, where nascent proteins interact with molecular chaperones (Saibil, 2013). Protein folding in the ER is directed by two major chaperone systems: these are the classical chaperone system and the carbohydrate‐binding system, although the two systems are not mutually exclusive (Braakman and Hebert, 2013). The folding of glycoproteins is intrinsically linked to N‐glycosylation (recently reviewed by Richard Strasser) (Strasser, 2018), as trimming of the glycan moiety signals entry and exit from the calnexin/calreticulin quality control cycle (Lannoo and Van Damme, 2015). Misfolded glycoproteins are retained in the ER and will cycle through the calnexin/calreticulin folding cycle until the correct conformation is achieved, or they will be targeted for degradation (Figure 3). In the absence of proper glycosylation, nascent glycoproteins may be prone to misfolding as a result of mispaired cysteine residues. This is best illustrated by the work of Daniels and colleagues, who provided evidence that the strategic placement of glycans on influenza HA serves to shield susceptible cysteine residues during folding (Daniels *et al*., 2003). Calnexin and calreticulin further form complexes with the oxidoreductase ERp57 which catalyses the formation of disulphide bonds (Frickel *et al*., 2004; Oliver *et al*., 1997, 1999; Zapun *et al*., 1998). In addition to ERp57, other members of the protein disulphide isomerase family are also responsible for catalysing disulphide bonds resulting in a level of built‐in redundancy into the system (Rutkevich *et al*., 2010).

Given the extended period of divergent evolution of mammals and plants, the low expression levels reported for many viral glycoproteins may be due to incompatibility with the endogenous plant chaperone machinery. In support of this, putative homologues of key chaperones known to interact with glycoproteins exhibit low‐level sequence identity to their human counterparts. We are currently exploring the co‐expression of human molecular chaperones *in planta* (patent application: PA167643/P) to improve the production of heterologous glycoproteins, based on the hypothesis that the endogenous plant chaperones may represent a critical bottleneck for high levels of expression.

The co‐expression of various protein disulphide isomerases may improve the disulphide bond formation of soluble HIV Env antigens produced in plants. The gp120 subunit of HIV Env contains nine intrachain disulphide bonds with an additional disulphide bond present in the transmembrane subunit (gp41) (Go *et al*., 2014; Leonard *et al*., 1990). Given the presence of 20 cysteine residues in each gp160 subunit, and the obvious potential for aberrant disulphide bond formation, reports of cross‐linked dimers and aggregates in the literature are unsurprising (Leonard *et al*., 1990; Owens and Compans, 1990). We and others have reported the presence of unresolved higher molecular weight products following Western blotting of plant‐produced HIV Env gp140 proteins (Rosenberg *et al*., 2013) (Margolin *et al*., submitted for publication). Rosenberg and colleagues speculated that these comprised of higher order oligomers that were incompletely resolved by SDS‐PAGE (Rosenberg *et al*., 2013). We hypothesize that these unresolved higher molecular weight products are actually aggregates of misfolded protein with scrambled disulphide bridges. We have observed that these higher molecular weight products do not dissociate in the presence of detergent or under varying conditions of pH and salt concentrations. However, boiling the recombinant proteins for extended lengths of time in the presence of β‐mercaptoethanol results in improved dissociation of these higher order molecular weight protein species, suggesting that improving the disulphide bond formation may improve protein production.

## Current candidate viral glycoproteins produced in plants

A number of viral glycoprotein vaccine candidates have been successfully expressed in plants to date; either transiently or by generation of transgenic or transplastomic plants (Table 2). These include antigens against high‐impact human diseases such as influenza and HIV, as well as those against vector‐borne and other zoonotic viral diseases such as Zika, dengue and West Nile fevers, Japanese encephalitis, coronavirus infections, Crimean–Congo haemorrhagic and Rift Valley fevers, rabies, infectious bronchitis, Newcastle disease and Ebola. Many of these have been tested for specific immunogenicity in animals, and some including infectious bronchitis, influenza, Newcastle disease and rabies have been tested for efficacy using animal challenge models, with encouraging results.

**Table 2:** Summary of recombinant glycoprotein vaccine antigens produced in plants

Virus	Glycoprotein (antigen)	Expression host (leaves unless otherwise specified)	Transient (T)/Transgenic (TG)/Transplastomic (TP) yields	Antigen tested in animals	Animal testing	Immunogenicity	Challenge efficacy	Reference
CCHFV	GnGc	*N. tabacum*	TG 0.9–1.4 mg/kg FW	Tobacco leaf pellets	BALB/c mice oral	IgG; IgA	ND	Ghiasi *et al*. (2011)
		*N. tabacum* hairy roots	TG 1.8 mg/kg FW	Hairy root pellets	BALB/c mice oral	IgG; IgA	ND	
Coronavirus	S1‐GFP fusion (his‐tagged)	*N. tabacum*	T NQ	NT	ND	–	–	Li *et al*. (2006)
		*N. tabacum*	TP 0.2% TSP	NT	ND	–	–	
	S1	*N. tabacum* root	TG NQ	Lyophilized tobacco root	ALB/c mice oral	IgG	ND	Pogrebnyak *et al*. (2005)
		*Lycopersicon esculentum* (tomato) fruit	TG NQ	Lyophilized tomato fruit	BALB/c mice oral	IgA	ND	
Dengue virus	pRM/E	*Lactuca sativa (lettuce)*	TP NQ	NT	ND	–	–	Kanagaraj *et al*. (2011)
	D2EIII	*N. benthamiana*	T 5.6 mg/kg FW	Ni+ affinity‐purified D2III	C3H mice IM	IgG and PRNT neutralization	ND	Saejung *et al*. (2007)
	Et (truncated)	*N. benthamiana*	T 600 mg/kg FW	NT	ND	–	–	Martinez *et al*. (2010)
	pRM/Et	*N benthamiana*	T 500 mg/kg FW	NT	ND	–	–	
	HBCag‐DIII	*N. benthamiana*	T 400 mg/kg FW	NT	ND	–	–	
	CTB‐EIII	*N. tabacum*	TG 0.0053%–0.019% TSP	NT	ND	–	–	Kim *et al*. (2010)
	EDIII (consensus)	*Oryza sativa (rice)* callus	TG 450 mg/kg FW	NT	ND	–	–	Kim *et al*. (2012)
Ebola virus	EIC (GP1 fusion with heavy chain monoclonal ab)	*N. benthamiana*	T 50 mg/kg FW	Protein G affinity‐purified EIC	BALB/c mice SC	IgG	ND	Phoolcharoen *et al*. (2011)
IBV	S (spike protein)	*Solanum tuberosum* (potato) tubers	TG 2.39–2.53 mg/kg FW	Fresh potato tuber; potato tuber extract	Chickens oral; IM	IL‐2; Neutralization	66.7% survival oral; 100% survival IM	Zhou *et al*. (2004)
JEV	EDIII‐BaMV CP	*N. benthamiana*	T NQ	NT	ND	–	–	Chen *et al*. (2017)
		*Chenopodium quinoa*	T 4300–8900 mg/kg	Purified chimeric virus particles	BALB/c mice IP	IgG; PRNT neutralization	ND	
HIV	gp140 ∆CFI Env	*N. benthamiana*	T 80 mg/kg FW	NT	ND	–	–	Rosenberg *et al*. (2013)
	dgp41 (deconstructed gp41)	*N. benthamiana*	T 9 mg/kg FW	NT	ND	–	–	Kessans *et al*. (2013)
	Consensus Env/HA chimaera	*N. benthamiana*	T NQ	NT	ND	–	–	WO 212/083445A1
	gp140 NFL	*N. benthamiana*	T 5–6 mg/kg	Lectin‐column‐purified gp140 NFL	Rabbits IM	Binding and neutralizing antibodies	ND	Margolin *et al*. (submitted for publication)
Influenza virus	H1N1 HA (haemagglutinin)	*N. benthamiana*	T 90 mg/kg FW	Ni+ affinity‐purified HA	Mice IM; rabbits IM; ferrets IM	HAI titres and virus neutralization	ND	Shoji *et al*. (2011)
	H5N1 HA	*N. benthamiana*	T 50 mg/kg FW	Ni+ affinity‐purified HA	Mice IM; rabbits IM; ferrets IM	HAI titres and virus neutralization	ND	
	H5N1 HA	*N. benthamiana*	T NQ	Ni+ affinity‐purified HA	BALB/c mice IN; ferrets IN	IgA, IgG; HAI titres and virus neutralization	Mice 100% survival; Ferrets 100% survival	Major *et al*. (2015)
	H5N1 HA (VLPs)	*N. benthamiana*	T 50 mg/kg FW	Fetuin affinity‐purified HA VLPs	BALB/c mice IM	HAI titres	100% protection	D'Aoust *et al*. (2008)
	H1N1 HA (VLPs)	*N. benthamiana*	T 50 mg/kg FW	Fetuin affinity‐purified HA VLPs	BALB/c mice IM	HAI titres	100% protection	
Newcastle disease virus	F (fusion protein)	*Zea mays* (maize) kernels	TG 0.9%–0.17% TSP	Ground kernels	Chicken oral	IgG	100% protection	Guerrero‐Andrade *et al*. (2006)
	HN	*Solanum tuberosum* tubers	TG 300–600 mg/kg	Crude leaf extracts	BALB/c mice IP; oral	IgG; IgG + IgA	ND	Berinstein *et al*. (2005)
	F	*S. tuberosum*	TG 300–600 mg/kg	Crude leaf extracts	BALB/c mice IP; oral	IgG; IgG + IgA	ND	
	HN	*N. benthamiana*	T 3000 mg/kg FW	NT	ND	–	–	Gomez *et al*. (2009)
Rabies virus	Surface protein G	*N. tabacum*	TG 0.1%–0.38% TSP	Human antirabies IgG affinity‐purified G	BALB/C mice IP	IgG	100% protection	Ashraf *et al*. (2005)
	Surface protein G	*Z. mays* kernels	TG 25 mg/kg FW	Ground kernels	Sheep oral	RFFIT neutralization	66% survival	Loza‐Rubio *et al*. (2012)
RVFV	Gn deletion mutant	*Arabidopsis thaliana* whole plants	TG NQ	Fresh leaves	C57/B1 mice oral	IgG	ND	Kalbina *et al*. (2016)
WNV	E domain III (DIII) (his‐tagged)	*N. benthamiana*	T 1.16–73 mg/kg FW	Ni+ affinity‐purified DIII	BALB/c mice SC	IgG	ND	He *et al*. (2014)
Zika virus	E glycoprotein (his‐tagged)	*N. benthamiana*	T 160 mg/kg FW	Ni+ affinity‐purified E	C57/BL6 mice SC	IgG; IFNƳ, IL‐4, IL6; PRNT neutralization	ND	Yang *et al*. (2017b)
	HBCag‐DIII (VLPs)	*N. benthamiana*	T 1.8 mg/kg FW	Sucrose gradient‐purified VLPs	C57/BL6 mice SC	IgG; IFNƳ; PRNT neutralization	ND	Yang *et al*. (2017a)

CCHFV, Crimean–Congo haemorrhagic fever virus; HAI, haemagglutinin inhibition; IBV, infectious bronchitis virus; IM, intramuscular; IN, intranasal; IP, intraperitoneal; JEV, Japanese encephalitis virus; mg/kg FW, mg/kg fresh weight; ND, not done; NQ, no quantitation; NT, not tested; PRNT, plaque reduction neutralization test; RFFIT, rapid fluorescent focus inhibition test; RVFV, Rift Valley fever virus; SC, subcutaneous; WNV, West Nile virus.

### Influenza

Human seasonal influenza disease is presently caused by three influenza virus A subtypes (H1N1, H1N1pdm09 and H3N2) and two influenza virus B subtypes (Yamagata and Victoria). Annually 5%–10% of adults and 20%–30% of children are infected, with 3–5 million cases of severe illness, and about 250 000–500 000 deaths (WHO, 2014), although this figure has recently been increased to 300 000–650 000 deaths, based on new modelling studies (Iuliano *et al*., 2017). The conventional chicken egg‐based vaccine technology has a production capacity as of 2011 of 1.4 billion doses of trivalent vaccine and a production level of 620 million doses. However, it takes 6 months to make and produce the vaccines, and thus, vaccine antigens are chosen based on the viruses circulating at that time, which in the Northern Hemisphere 2017–2018 influenza season has been predicted to cause problems due to H3N2 vaccine mismatch with the circulating type (Dugan, 2017).

The potential for rapid response and scalability of plant‐based expression is best illustrated by the pioneering work of Medicago Inc. and the Fraunhofer USA group in the development of candidate vaccines for pandemic influenza strains (D'Aoust *et al*., 2008; Shoji *et al*., 2011; Stoger *et al*., 2014; Streatfield *et al*., 2015). Given the slow production time and limited production capacity of using embryonated hen's eggs, traditional influenza vaccine manufacturers are not equipped to respond timeously, or in sufficient volume, to a pandemic outbreak (D'Aoust *et al*., 2010; Quan *et al*., 2016). Typical egg‐based production of split virion vaccines can take up to 6 months due to the limitations inherent in the production platform which can be further hampered by variable production yields (Manini *et al*., 2017). In contrast, Medicago Inc in the USA has reported the production of a fully formulated HA VLP vaccine within 21 days from the release of the sequence for the pandemic A/H1N1(A/California/04/09) strain (D'Aoust *et al*., 2010). Furthermore, their production platform is rapidly scalable and has been used to successfully produce 10 million doses of a monovalent H1N1 VLP vaccine within 30 days under the Defense Advanced Research Projects Agency (DARPA) Blue Angel programme [https://www.rt.com/usa/future-vaccine-darpa-research-255/].

The Fraunhofer Centre for Molecular Biotechnology (USA) has described the development of a similar transient plant expression platform for the production of soluble HA antigens which has been reported to yield purified vaccines in just over a month (Shoji *et al*., 2012). Both of these platforms are highly flexible and have been used to produce HA from several seasonal and pandemic influenza subtypes (D'Aoust *et al*., 2008; Mett *et al*., 2008; Pillet *et al*., 2015; Shoji *et al*., 2008, 2009a,b, 2011, 2012). These antigens have demonstrated promising immunogenicity in preclinical animal models with several reports of protection against lethal virus challenge (D'Aoust *et al*., 2008; Mett *et al*., 2008; Pillet *et al*., 2015; Shoji *et al*., 2008, 2009a,b, 2011). Consistent with these studies, clinical trials have reported that these vaccines are safe and highly immunogenic in human volunteers (Chichester *et al*., 2012; Cummings *et al*., 2014; Landry *et al*., 2010, 2014; Ward *et al*., 2014). Prompted by the limited access to vaccines in South Africa during the 2009 H1N1 pandemic, our group has developed a similar production platform for the expression of both full‐length and soluble HA antigens (Mortimer *et al*., 2012).

### HIV

The human immunodeficiency virus is responsible for an unprecedented global pandemic, particularly in sub‐Saharan Africa which bears a disproportionate burden of global infections relative to its size (Shao and Williamson, 2012). A broadly protective vaccine is urgently needed to combat the spread of HIV, and even a partially effective vaccine is expected to make a large impact on the epidemic (Medlock *et al*., 2017). Current state‐of‐the‐art subunit vaccines are based on rationally engineered soluble Env trimer mimetics produced in mammalian cell culture systems, with the intention of eliciting neutralizing antibodies (Sanders and Moore, 2017). The high costs and limited production capacity available are expected to be particularly problematic, considering the likelihood that repeat immunizations will be required (Shao and Williamson, 2012). Given the structural similarity between influenza HA and the HIV Env glycoprotein, it is reasonable to assume that plants may have the capacity to emulate the structure of the HIV Env glycoprotein trimer (Karlsson Hedestam *et al*., 2008). This has prompted us to explore the potential of *N. benthamiana* for the production by transient agroinfection of trimeric Env glycoproteins based on HIV subtypes circulating in sub‐Saharan Africa (patent application: PPA UK 1617480.7; Margolin *et al*., submitted for publication).

Very few studies have reported the successful expression of a significant portion of the HIV Env glycoproteins in plants. The first published study describing the expression of the majority of the HIV Env glycoprotein was by Rosenberg and colleagues, who produced a soluble gp140 protein (protein lacks the transmembrane and cytoplasmic domains) in *N. benthamiana* as a reagent to evaluate the reactivity of plant‐produced monoclonal antibodies (Rosenberg *et al*., 2013). The authors reported that the recombinant Env protein was produced at ~80 mg/kg by both transient and stable transgenic expression (Rosenberg *et al*., 2013). Preceding this, a patent filed by Medicago Inc. described the production of chimeric virus‐like particles comprising of a synthetic consensus group M Env protein fused to the transmembrane and cytoplasmic domains of influenza HA. The patent application further reported that expression of the native HIV Env protein could not be detected (international patent application number: WO 2012/083445 A1). The antigens produced in both of these studies were modified to remove the cleavage site which is now known to result in an aberrant structure, unless a flexible linker peptide is introduced at the interface of the gp120 and gp41 to retain the conformational mobility of the two subunits (Georgiev *et al*., 2015; Ringe *et al*., 2013; Sharma *et al*., 2015). It is also noteworthy that the major surface glycoprotein from simian immunodeficiency virus (SIV) (gp130) has been successfully produced in transgenic corn with the aim of developing an orally immunogenic vaccine, although no immunogenicity was reported (Horn *et al*., 2003).

We have recently described the development of a platform for the expression of soluble HIV‐1 Env gp140 trimers in *N. benthamiana* plants (patent application: PPA UK 1617480.7). These antigens exploit a 10 residue glycine‐rich linker peptide at the interface of the gp120 and gp41 subunits to eliminate the requirement for furin‐mediated cleavage, which does not naturally occur in plants (Wilbers *et al*., 2016). Preliminary immunogenicity studies confirmed that the proteins were immunogenic in rabbits; however, despite eliciting high titres of binding antibodies, the vaccines only induced low levels of neutralizing antibodies. The removal, by size exclusion chromatography, of nontrimeric Env species improved the induction of neutralizing antibodies which improved further when animals were primed with recombinant modified vaccinia Ankara (MVA) virus expressing Gag and Env antigens. To our knowledge, this is the first study to report the immunogenicity of a plant‐produced HIV‐1 Env trimer in an animal model (Margolin *et al*., submitted for publication).

### Flaviviruses

The flaviviruses West Nile (WNV), Zika (ZIKV), yellow fever (YFV), Japanese encephalitis (JEV) and dengue (DENV) comprise a group of specifically mosquito‐borne viruses, within the family *Flaviriridae*. The recent emergence and re‐emergence of WNV, YFV and ZIKV in the Americas (Zumla *et al*., 2016), and of WNV and tick‐borne encephalitis in Europe (Kaaijk and Luytjes, 2017), have resulted in renewed interest in the development of prophylactic vaccines against these pathogens. These concerns have been further compounded by the finding that infection with ZIKV during pregnancy was associated with birth defects during a recent outbreak in Brazil (Yakob and Walker, 2016). Similar limitations in terms of the scalability and production costs of conventional production platforms as for influenza vaccines have driven interest in the potential of molecular farming to produce vaccines for these emerging flaviruses (Cardona‐Ospina *et al*., 2016; He *et al*., 2014; Yap and Smith, 2010). Several dengue envelope antigens have been produced in plants, although there is a paucity of immunogenicity data to support their utility as vaccine immunogens (Kim *et al*., 2010, 2012; Martinez *et al*., 2010; Saejung *et al*., 2007).

The domain III of the flavivirus Env glycoprotein is targeted by neutralizing antibodies during natural infection and is therefore an important target for vaccine‐mediated protection (Beasley and Barrett, 2002; Crill and Roehrig, 2001; Zhao *et al*., 2016). Martínez and colleagues have reported the successful production of both a truncated envelope protein and a chimeric fusion between the hepatitis B core antigen and the dengue virus envelope domain III (EDIII), for potential use as a vaccine or diagnostic reagent (Martinez *et al*., 2010). Dengue EDIII has also been produced as a fusion protein with *Vibrio cholera* toxin B subunit in transgenic potatoes—presumably for use as an oral vaccine, although no immunogenicity was reported (Kim *et al*., 2010). Similarly, EDIII has been expressed as a soluble protein in both transgenic tobacco and rice callus. The dengue virus glycoprotein has also been produced as a modified polyprotein in transplastomic lettuce, containing part of the capsid in addition to the premembrane and Env proteins (Kanagaraj *et al*., 2011). In spite of the lack of immunogenicity reports for many of these studies, immunization of mice with *N. benthamiana*‐derived dengue EDIII has been shown to elicit neutralizing antibodies in mice, warranting further development of these antigens (Saejung *et al*., 2007). Similarly, a transiently expressed West Nile virus EDIII antigen was reported to be immunogenic in mice (He *et al*., 2014). Chimeric bamboo mosaic virus particles presenting EDIII from Japanese encephalitis virus have also been produced in plants and were shown to elicit neutralizing antibodies in mice (Chen *et al*., 2017). More recently, increases in demand and reports of adverse effects against the current live attenuated YFV vaccine have prompted the development of a plant‐produced subunit vaccine as an alternative (Tottey *et al*., 2018). Tottey and colleagues successfully produced the envelope glycoprotein, as a soluble protein and as a chimeric fusion to bacterial lichenase, both of which protected mice from lethal challenge. It should be noted, however, that these were comparatively less immunogenic than the 17DD live attenuated vaccine (Tottey *et al*., 2018). Many of these soluble vaccine candidates would benefit from multimerization in the form of synthetic nanoparticles to improve their immunogenicity. The large size of nanoparticles facilitates their entry into lymphatics and enables drainage to lymph nodes to interact with professional antigen‐presenting cells (Manolova *et al*., 2008). In addition, repeating arrays of antigens promote the cross‐linking of B cells, resulting in sustained and durable antibody responses even in the absence of T‐cell stimulation (Bachmann and Jennings, 2010; Zabel *et al*., 2013).

Motivated by the recent outbreaks in the Americas, two studies from Arizona State University have described the production of Zika virus glycoprotein antigens in plants. The first successfully expressed a soluble E protein which retained native antigenicity based on the binding of a panel of monoclonal antibodies and elicited both cellular and humoral responses in immunized mice. Furthermore, the levels of neutralizing antibodies in immunized animals exceeded the threshold required for protective immunity (Yang *et al*., 2017b). The same group has also developed a VLP vaccine comprised of the hepatitis B core antigen presenting the Zika virus E protein domain III, to circumvent potentially inducing antibody‐dependent enhancement (ADE) of infection against dengue infection (Yang *et al*., 2017a).

### Other vector‐borne and zoonotic viruses

Several vector‐borne and zoonotic diseases have recently been identified as prime candidates for the development of vaccines and diagnostics under the One Health initiative: notable examples are the bunyaviruses Crimean–Congo haemorrhagic fever and Rift Valley fever viruses (Rybicki, 2017). Many of these pathogens affect livestock and cause zoonotic infections through direct contact with humans. It is also worth noting that the less stringent regulatory requirements for the commercialization of veterinary vaccines make for a comparatively quicker road to market than human vaccines—and that a successful animal vaccine could quickly become a human vaccine for these viruses (Rybicki, 2010). To this end, a soluble RVFV Gn glycoprotein has been expressed at low levels in stably transformed *Arabidopsis thaliana*. Although the recombinant protein could not be detected by Western blotting, mRNA expression was confirmed by RT‐PCR and fresh plant material was reported to be orally immunogenic in mice (Kalbina *et al*., 2016). Our group has expressed and purified a chimeric particle‐forming Gn antigen from *N. benthamiana* plants which contains the transmembrane and cytoplasmic domains of influenza HA (Mbewana, 2017). This vaccine candidate is of particular interest as immunity against Gn is sufficient to confer protective immunity (de Boer *et al*., 2010). The full‐length Crimean–Congo haemorrhagic fever virus GnGc protein has also been successfully produced in tobacco leaves and hairy root cultures. When mice were fed with the transgenic material, both IgG and IgA antibodies were elicited, although expression levels of the viral proteins were very low (Ghiasi *et al*., 2011).

Another plant‐produced viral glycoprotein with potential as a vaccine antigen is the rabies virus glycoprotein G, which has been expressed in transgenic maize, tobacco and tomatoes (Ashraf *et al*., 2005; Loza‐Rubio *et al*., 2008; McGarvey *et al*., 1995). Transgenic maize expressing the G protein was shown to protect sheep from virulent challenge, although the level of protection was lower than the commercial vaccine (Loza‐Rubio *et al*., 2012). The full‐length glycoprotein from infectious bronchitis virus (IBV) glycoprotein has also been produced in transgenic potatoes and tuber extract was sufficient to protect chickens against virulent challenge following both oral and intramuscular immunizations (Zhou *et al*., 2004). Similarly, an orally immunogenic Newcastle disease virus fusion protein produced in transgenic maize was reported to protect chickens against challenge (Guerrero‐Andrade *et al*., 2006).

The extracellular subunit of the Ebola virus glycoprotein (GP1) has been expressed in tobacco plants as an immune complex with a human monoclonal antibody (Phoolcharoen *et al*., 2011). Interestingly, the authors reported that GP1 could not be produced in sufficient quantities without the heavy chain fusion, due to extensive leaf necrosis. They hypothesized that the presence of the monoclonal antibody segment enabled improved recruitment of molecular chaperones, once again suggesting that the plant chaperone machinery may represent a critical bottleneck for glycoprotein expression. The GP1 component of the fusion protein was properly folded, based on reactivity of a conformational monoclonal antibody. The authors further reported that subcutaneous immunization of mice with the immune complex elicited immune responses comparable to a protective Venezuelan equine encephalitis virus vaccine, although the antibody titres were admittedly lower. A truncated form of the SARS Coronavirus spike glycoprotein has been expressed by transplastomic and stable nuclear transformation of both tobacco and lettuce leaves, for use as an oral vaccine (Li *et al*., 2006).

## Conclusion

Molecular farming is on the cusp of being accepted as a mainstream technology for the production of heterologous pharmaceuticals, with particular appeal for resource‐limited regions. Recent years have borne witness to the licensure of the first plant‐made pharmaceutical for use in humans—taliglucerase alpha—with several more presently advancing through clinical trials. Plant‐made pharmaceuticals have proven to be highly scalable and safe in humans, despite unfounded concerns regarding the impact of plant‐specific glycosylation. Limitations of expression yields have been addressed with improved expression vectors enabling high levels of expression of many pharmaceutically relevant proteins.

Although historically glycoproteins have been difficult to produce *in planta*, a number of different viral glycoproteins have been successfully expressed in plants, including most notably influenza HA and HIV Env, and several glycoproteins from emerging pathogens (D'Aoust *et al*., 2008). Fundamental differences in plants have been addressed by the manipulation of the secretory pathway, such as by glycoengineering, or the co‐expression of various other proteins to modify the host cell environment (Goulet *et al*., 2012; Jutras *et al*., 2015; Steinkellner and Castilho, 2015). Whilst many of the considerations discussed in this review are important for the production of viral glycoproteins in plants, it is likely that additional bottlenecks still remain and that these bottlenecks may even vary for different viral glycoproteins. Once these factors are identified, appropriate strategies can be conceived to expand the range of viral glycoproteins produced *in planta*. In conclusion, the use of molecular farming for the production of viral glycoproteins is an exciting and rapidly growing application of the technology with the potential to provide urgently needed vaccines and diagnostics for regions where they are needed the most.

## Conflict of interest

The authors have filed patent applications protecting the production of HIV envelope trimers in plants (PA166256P) and the co‐expression of human molecular chaperones in plants to improve the production of heterologous proteins (PA167643_P).
